# mstree: A Multispecies Coalescent Approach for Estimating Ancestral Population Size and Divergence Time during Speciation with Gene Flow

**DOI:** 10.1093/gbe/evaa087

**Published:** 2020-05-04

**Authors:** Junfeng Liu, Qiao Liu, Qingzhu Yang

**Affiliations:** e1 Beijing Institute of Genomics, Chinese Academy of Sciences, Beijing, China; e2 Department of Automation, Tsinghua University, Beijing, China

**Keywords:** coalescent, gene tree, mathematical inequalities

## Abstract

Gene flow between species may cause variations in branch length and topology of gene tree, which are beyond the expected variations from ancestral processes. These additional variations make it difficult to estimate parameters during speciation with gene flow, as the pattern of these additional variations differs with the relationship between isolation and migration. As far as we know, most methods rely on the assumption about the relationship between isolation and migration by a given model, such as the isolation-with-migration model, when estimating parameters during speciation with gene flow. In this article, we develop a multispecies coalescent approach which does not rely on any assumption about the relationship between isolation and migration when estimating parameters and is called mstree. mstree is available at https://github.com/liujunfengtop/MStree/ and uses some mathematical inequalities among several factors, which include the species divergence time, the ancestral population size, and the number of gene trees, to estimate parameters during speciation with gene flow. Using simulations, we show that the estimated values of ancestral population sizes and species divergence times are close to the true values when analyzing the simulation data sets, which are generated based on the isolation-with-initial-migration model, secondary contact model, and isolation-with-migration model. Therefore, our method is able to estimate ancestral population sizes and speciation times in the presence of different modes of gene flow and may be helpful to test different theories of speciation.

## Introduction

The role of gene flow in speciation is a fundamental issue in evolutionary biology. Allopatric speciation considers complete lack of gene flow as prerequisite to the formation of new species. However, parapatric and sympatric speciation allow gene flow during speciation. Although allopatric speciation has been historically taken as the paramount mode of speciation (Futuyma and Mayer 1980), theoretical modeling and empirical evidence increasingly support that speciation can occur with gene flow (Gourbiere and Mallet 2010; Smadja and Butlin 2011; Feder et al. 2012).

There are usually two kinds of models to make inferences about gene flow during speciation. Some methods are based on an isolation-with-migration (IM) model (Wang and Hey 2010; Tian and Kubatko 2016; Dalquen et al. 2017) and others on an isolation-with-initial-migration (IIM) model (Mailund et al. 2012; Costa and Wilkinson-Herbots 2017). However, the above two models include an assumption about the relationship between isolation and migration. Here, we use the properties of coalescent-based model in gene tree data for estimating the important parameters such as ancestral population sizes and divergence times without any assumption of the relationship between isolation and migration. Furthermore, we conduct simulations to examine the accuracy of the estimates of parameters. The simulation results show that our method can accurately estimate the parameters. At last, we compared mstree with the program 3s (Dalquen et al. 2017) and IMa3 (Hey et al. 2018) with simulation data; the simulation results show that mstree is faster than 3s and IMa3.

## Materials and Methods

### The Theoretical Model

**Fig. 1.**
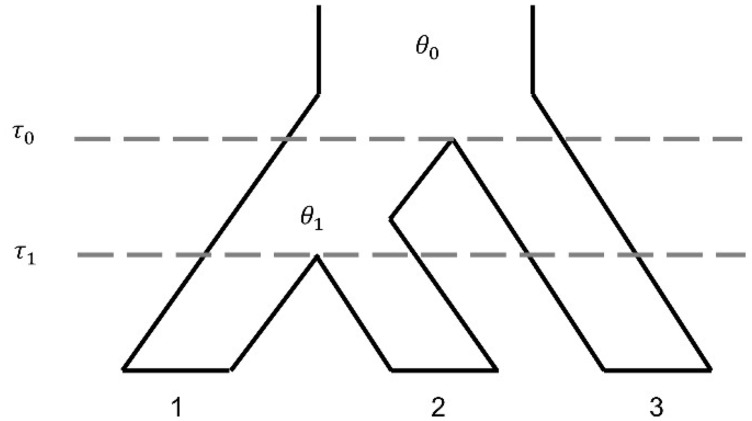
—Species tree ((1, 2), 3) for three species. The species divergence times are denoted as $\tau_{0}$ and $\tau_{1}$. The ancestral species population sizes are denoted as $\theta_{0}$ and $\theta_{1}$.

**Fig. 2.**
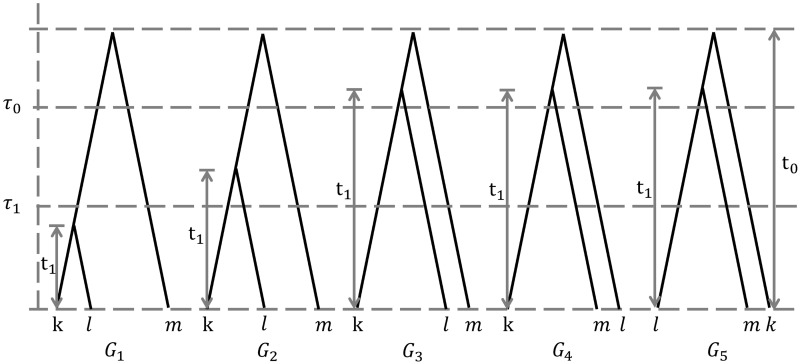
—For three species (1–3) with gene flow between species 1 and 2, there are five categories of gene trees for any locus with three sequences (*k*, *l*, and *m*), which are from species 1, species 2, and species 3, respectively.

Consider two closely related species (1 and 2) with an outgroup species 3. We assume that there is only gene flow between two closely related species (fig. 1). We use $\tau_{0}$ and $\tau_{1}$ to denote the two species divergence times, scaled by mutation rate. Let $\theta_{0}=4N_{0}\mu$ and $\theta_{1}=4N_{1}\mu$ measure the two ancestral species population sizes. Here, μ is the mutation rate per site and generation, and the $N_{0}$ and $N_{1}$ denote the effective population sizes. There are five possible gene trees for a locus with three sequences (*k*, *l*, and *m*), which are from species 1, species 2, and species 3, respectively (fig. 2). For any locus, $t_{0}$ is the coalescent time among three sequences and $t_{1}$ is the coalescent time between two sequences. In the presence of gene flow between species 1 and species 2, the gene tree *G*_1_ may be possible at a locus. Otherwise, only gene trees *G*_2_–*G*_5_ are possible. In this study, the term “loci” refers to independent or loosely linked short segments of the genome, and we assume that there is no recombination within a locus while different loci are free recombining. For tens of thousands of loci, there are some mathematical inequalities among the species divergence time, the ancestral species population size, and the number of gene trees, of which $t_{1}$ is larger than $\tau_{1}$. Based on the coalescent theory with no gene flow under given species, the probability of gene tree, of which $t_{1}$ belongs to $\left[\tau_{1,}\right.\left.\tau_{0}\right)$, is $1-e^{-2\left(\tau_{0}-\tau_{1}\right)/\theta_{1}}$; and the probability of gene tree, of which $t_{0}$ belongs to $\left[\tau_{0}\right.\left.{,\tau }_{0}^{'}\right)$ and $t_{1}$ is less than $\tau_{0}$, is $1-e^{-2\left(\tau_{0}^{'}-\tau_{0}\right)/\theta_{0}}$. We use *g_i_*([*a*, *b*],[*c*, *d*]) as the number of gene trees with category *G_i_* (fig. 2) and with $t_{0}$ is in [*a*, *b*] and $t_{1}$ in [*c*, *d*] for *i* = 1, 2, …, 5. Moreover, to simplify notation, let *g_i_* denote the number of gene trees with category *G_i_* (fig. 2) for *i* = 1, …, 5. Then, the formulas of cases A and C are as follows:

Case A: If ${\tau_{1}\leq \tau }_{1}^{'}<\tau_{0}$, then $\frac{g_{3}+g_{4}+g_{5}}{g_{2}(\left[\tau_{0},\infty \right],[\tau_{1}^{'},\tau_{0}])+g_{3}+g_{4}+g_{5}}\approx e^{-2\left(\tau_{0}-\tau_{1}^{'}\right)/\theta_{1}}$ for the category *G*_2_–*G*_5_ (fig. 2).

Case B: If $\tau_{0}\leq \tau_{0}^{'}<\tau_{0}^{''}$, then $\frac{g_{1}\left(\left[\tau_{0}^{''},\infty \right],\left[0,\tau_{0}\right]\right)+g_{2}(\left[\tau_{0}^{''},\infty \right],[0,\tau_{0}])}{g_{1}\left(\left[\tau_{0}^{'},\infty \right],\left[0,\tau_{0}\right]\right)+g_{2}(\left[\tau_{0}^{'},\infty \right],[0,\tau_{0}])}\approx e^{-2\left(\tau_{0}^{''}-\tau_{0}^{'}\right)/\theta_{0}}$ for the category *G*_1_–*G*_2_ (fig. 2).

Case C: $\frac{g_{4}+g_{5}}{{g_{2}+g_{3}+g}_{4}+g_{5}}\approx {\frac{2}{3}e}^{-2\left(\tau_{0}-\tau_{1}\right)/\theta_{1}}$ for the category *G*_2_–*G*_5_ (fig. 2).

The above gene tree distributions allow us to compute the parameters $\theta_{0}$, $\tau_{0}$, $\theta_{1}$, and $\tau_{1}$. The approach of estimating the parameters is called mstree and the strategies are as follows. First, we can estimate the value of $\tau_{0}$ based on the fact that the shape of gene tree may be ((*k*, *l*),*m*), ((*k*, *m*),*l*) or ((*l*, *m*),*k*) with equal probability when $t_{1}$ is larger than $\tau_{0}$. If there exists $t^{'}$ that satisfies $2a\approx n_{2}+n_{3}$, where a is the number of gene trees, of which $t_{1}$ is larger than $t^{'}$ and the shape is ((*k*, *l*),*m*) (fig. 2: *G*_3_); $n_{2}$ is the number of gene trees, of which the shape is ((*k*, *m*),*l*) (fig. 2: *G*_4_); and $n_{3}$ is the number of gene trees, of which the shape is ((*l*, *m*),*k*) (fig. 2: *G*_5_), $t^{'}$ can be considered as the estimated value of $\tau_{0}$. Second, we can estimate the value of $\theta_{0}$ based on the estimated value of $\tau_{0}$ by using the formula $e^{-2\left(\tau_{0}^{''}-\tau_{0}^{'}\right)/\theta_{0}}\approx \frac{a}{a+b^{'}}$ in case B. When we choose $t^{'}$ and $t^{''}$ that satisfy $\tau_{0}\leq t^{'}<t^{''}$, the estimated value of $\theta_{0}$ approximates $-2\left(t^{''}-t^{'}\right)/\log\left(a/a+b^{'}\right)$, where a is the number of gene trees, of which $t_{0}$ is larger than $t^{''}$ and $t_{1}$ is less than $\tau_{0}$; $b^{'}$ is the number of gene trees, of which $t_{0}$ belong to $\left[t^{'},\right.\left.t^{''}\right)$ and $t_{1}$ is less than $\tau_{0}$. Similarly, we can also estimate the value of $\theta_{1}$ based on the estimated value of $\tau_{0}$ by using the formula $e^{-2\left(\tau_{0}-\tau_{1}^{'}\right)/\theta_{1}}\approx \frac{a}{a+b^{'}}$ in case A. If we choose $t^{'}$ that is less than $\tau_{0}$ and assume that $t^{'}$ is larger than $\tau_{1}$ when $t^{'}$ is closed to $\tau_{0}$, the estimated value of $\theta_{1}$ approximates $-2\left(\tau_{0}-t^{'}\right)/\log\left(a/a+b^{'}\right)$, where a is the number of gene trees, of which $t_{1}$ is larger than $\tau_{0}$ and $b^{'}$ is the number of gene trees, of which $t_{1}$ belongs to $\left[t^{'},\right.\left.\tau_{0}\right)$. Lastly, we estimate the value of $\tau_{1}$ based on the values of $\tau_{0}$ and $\theta_{1}$ by using the formula ${\frac{2}{3}e}^{-2\left(\tau_{0}-\tau_{1}\right)/\theta_{1}}\approx \frac{n_{2}+n_{3}}{a+n_{2}+n_{3}}$ in case C. If there exists $t^{'}$ that is less than $\tau_{0}$ and satisfies ${\frac{2}{3}e}^{-2\left(\tau_{0}-t^{'}\right)/\theta_{1}}\approx \frac{n_{2}+n_{3}}{a+n_{2}+n_{3}}$, where a is the number of gene trees, of which $t_{1}$ is larger than $t^{'}$ and the shape is ((*k*, *l*),*m*) (fig. 2: *G*_2_–*G*_3_); $n_{2}$ is the number of gene trees, of which the shape is ((*k*, *m*),*l*) (fig. 2: *G*_4_); and $n_{3}$ is the number of gene trees, of which the shape is ((*l*, *m*),*k*) (fig. 2: *G*_5_), $t^{'}$ can be considered as the estimated value of $\tau_{1}$.

### The Simulation

We simulated gene trees by using the program ms (Hudson 2002) and converted gene trees to sequence data under JC69 model by using seq-gen (Rambaut and Grassly 1997). The example of command is as follows:

./ms id="465" 3 50000 -T -I 3 1 1 1 -m 1 2 0 -m 2 1 0 -em 0.667 1 2 4 -em 0.667 2 1 4 -em 1 1 2 0 -em 1 2 1 0 -ej 1 2 1 -ej 2 3 1 | tail -n + 4 | grep -v//> tree

./seq-gen -m HKY -l 500 -s 0.01 -t 2.0 < tree > infile

### Compared with IMa3 and Analyzed Real Data

The model estimated by IMa3 is IM model, and we used a fixed true species topology for IMa3. The real data are the genomic sequences of the human (H), chimpanzee (C), and gorilla (G) from Burgess and Yang (2008). The data set comprises 14,663 autosomal loci, and the mean locus length is 508 bp.

## Results

### The Accuracy of mstree

We used the program ms (Hudson 2002) to simulate gene trees at multi loci under the IIM, secondary contact (SC), and IM model. For the IIM model, the gene flow stopped at ${\frac{2}{3}\tau }_{1}$ in the past; For the SC model, the time of SC began at ${\frac{1}{3}\tau }_{1}$ in the past. Two sets of parameter values were used, roughly based on estimates from the hominoids (Burgess and Yang 2008) and the mangroves (Zhou et al. 2007). They are as follows: ${\theta_{0}=\theta }_{1}=0.005$, $\tau_{0}=0.006$, and $\tau_{1}=0.004$ (hominoids); ${\theta_{0}=\theta }_{1}=0.01$, $\tau_{0}=0.02$, and $\tau_{1}=0.01$ (mangroves). For the three models, gene flow is symmetrical and the migration rate (the expected number of migrants per generation) is 1. The number of loci is 10,000 and the number of replicates is 1,000. Analyzing the simulation data by using mstree, the results show that the parameter estimates are very close to the true values and are not sensitive to the model’s assumption about the relationship between isolation and migration (table 1). ε in table 1 is the threshold value in mstree and describes the degree of approximation between two sides of the formulas in cases A, B, and C. For example, in case A, ε=0.03 means the value of $\left|e^{-2\left(\tau_{0}-\tau_{1}^{'}\right)/\theta_{1}}-\frac{a}{a+b^{'}}\right|/\left(\frac{a}{a+b^{'}}\right)$ should be <0.03 when $e^{-2\left(\tau_{0}-\tau_{1}^{'}\right)/\theta_{1}}\approx \frac{a}{a+b^{'}}$. In mstree, the value of ε must be <0.05. The results in table 1 show that the smaller ε increased the standard deviations of the parameter estimates and the estimate of $\tau_{1}$. Therefore, we suggest that the value of ε should be 0.03 when using mstree. Furthermore, we applied mstree to additional two parameter sets (Dalquen et al. 2017), which have larger parameter values and different values for two θs (table 2). The results show that mstree still performs well.

**Table 1:** The Estimated Species Divergence Time and Population Size with Different Threshold Value

	Threshold	Hominoid				Mangrove			
		$\theta_{0}$	$\theta_{1}$	$\tau_{0}$	$\tau_{1}$	$\theta_{0}$	$\theta_{1}$	$\tau_{0}$	$\tau_{1}$
IIM model	*ε* = 0.007	0.50 ± 0.02	0.49 ± 0.04	0.60 ± 0.00	0.42 ± 0.04	1.00 ± 0.06	0.97 ± 0.11	2.00 ± 0.01	1.25 ± 0.29
	*ε* = 0.01	0.50 ± 0.02	0.49 ± 0.03	0.60 ± 0.00	0.41 ± 0.03	1.00 ± 0.05	0.98 ± 0.07	2.00 ± 0.01	1.14 ± 0.22
	*ε* = 0.03	**0.50** ± **0.02**	**0.50** ± **0.01**	**0.60** ± **0.00**	**0.39** ± **0.01**	**1.00** ± **0.02**	**0.99** ± **0.02**	**2.00** ± **0.01**	**0.99** ± **0.05**
SC model	*ε* = 0.007	0.50 ± 0.02	0.48 ± 0.05	0.60 ± 0.00	0.43 ± 0.05	1.00 ± 0.06	0.95 ± 0.14	2.00 ± 0.01	1.31 ± 0.33
	*ε* = 0.01	0.50 ± 0.01	0.49 ± 0.04	0.60 ± 0.00	0.42 ± 0.03	1.00 ± 0.04	0.97 ± 0.09	2.00 ± 0.01	1.21 ± 0.27
	*ε* = 0.03	**0.50** ± **0.01**	**0.50** ± **0.02**	**0.60** ± **0.00**	**0.39** ± **0.01**	**1.00** ± **0.02**	**0.99** ± **0.03**	**2.00** ± **0.01**	**1.01** ± **0.07**
IM model	*ε* = 0.007	0.50 ± 0.02	0.48 ± 0.06	0.60 ± 0.00	0.43 ± 0.05	1.00 ± 0.06	0.95 ± 0.15	2.00 ± 0.01	1.34 ± 0.34
	*ε* = 0.01	0.50 ± 0.01	0.49 ± 0.04	0.60 ± 0.00	0.41 ± 0.04	1.00 ± 0.04	0.96 ± 0.11	2.00 ± 0.01	1.22 ± 0.28
	*ε* = 0.03	**0.50** ± **0.01**	**0.50** ± **0.02**	**0.60** ± **0.00**	**0.39** ± **0.01**	**1.00** ± **0.02**	**0.99** ± **0.03**	**2.00** ± **0.01**	**1.00** ± **0.07**

Note.—The hominoid set is *θ*_0_ = *θ*_1_ = 0.005, *τ*_0_ = 0.006, and *τ*_1_ = 0.004. The mangrove set is *θ*_0_ = *θ*_1_ = 0.01, *τ*_0_ = 0.02, and *τ*_1_ = 0.01. *θ* and *τ* estimates are scaled by 10^2^. Gene flow is symmetrical and the migration rate is 1. *ε* is the threshold value in mstree. The number of loci is 10,000. The number of replicates is 1,000. IIM, isolation-with-initial-migration; SC, secondary contact; IM, isolation-with-migration. The best estimates are marked in bold.

**Table 2:** The Estimated Species Divergence Time and Population Size with Larger and Different Parameter Values

	Threshold	*θ*_0_ = 0.02, *θ*_1_ = 0.03, *τ*_0_ = 0.06, *τ*_1_ = 0.04				*θ*_0_ = 0.02, *θ*_1_ = 0.01, *τ*_0_ = 0.02, *τ*_1_ = 0.01			
		$\theta_{0}$	$\theta_{1}$	$\tau_{0}$	$\tau_{1}$	$\theta_{0}$	$\theta_{1}$	$\tau_{0}$	$\tau_{1}$
IIM model	*ε* = 0.03	2.00 ± 0.08	2.97 ± 0.10	5.99 ± 0.03	3.90 ± 0.17	2.00 ± 0.06	1.00 ± 0.02	2.00 ± 0.01	1.00 ± 0.03
SC model	*ε* = 0.03	2.01 ± 0.06	2.95 ± 0.17	5.99 ± 0.03	4.03 ± 0.19	2.00 ± 0.06	1.00 ± 0.02	2.00 ± 0.02	1.00 ± 0.04
IM model	*ε* = 0.03	2.00 ± 0.05	2.94 ± 0.29	5.98 ± 0.05	3.89 ± 0.46	2.00 ± 0.05	1.00 ± 0.02	2.00 ± 0.02	1.00 ± 0.04

Note.—*θ* and *τ* estimates are scaled by 10^2^. Gene flow is symmetrical and the migration rate is 1. *ε* is the threshold value in mstree. The number of loci is 10,000. The number of replicates is 1,000. IIM, isolation-with-initial-migration; SC, secondary contact; IM, isolation-with-migration.

### The Factors That Influence Parameter Estimates

In addition, we performed more simulations to test how different factors influence parameter estimates, such as the number of loci, migration rate, and the direction of migration. The numbers of loci are 5,000, 10,000, and 50,000; the migration rates are 0.1, 1, and 10; and the directions of migration are symmetrical and asymmetrical. The results are shown in supplementary tables S1–S6, Supplementary Material online. For the parameters $\theta_{0}$, $\theta_{1}$, and $\tau_{0}$, the results show that larger number of loci makes the estimates more accurate and the estimates are not sensitive to the model’s assumption, migration rate, and the direction of migration. For the parameter $\tau_{1}$, we have the same conclusion except for the case that migration rate is 10. When migration rate is 10, asymmetrical gene flow decreases the accuracy of $\tau_{1}$ estimate. This indicates that the estimate of $\tau_{1}$ is sensitive to the direction of migration with large migration rate (supplementary fig. S1, Supplementary Material online). The examples of above simulation commands are in the supplementary file S2, Supplementary Material online.

### Compared with 3s and IMa3

The input file of mstree is gene tree, which is in Newick format, and the gene tree can be estimated from the observed sequence alignments where there must be three sequences, with one sequence from each species, at each locus. Therefore, we need a program, such as PHYLIP, to infer gene trees when applying mstree to experimental data. Because inference of gene trees is associated with error and uncertainty, we did some simulations to investigate the effect of the gene tree uncertainty (supplementary table S7, Supplementary Material online). We used program ms and seq-gen (Rambaut and Grassly 1997) to generate sequence data under JC69 model and used program dnamlk in PHYLIP package to infer gene trees. Although there has been some decline in the accuracy of parameter estimates because of the inferred error of gene trees, the estimates of mstree are still near to the true values and not sensitive to the model’s assumption. Comparing mstree with the program 3s (Dalquen et al. 2017) and IMa3 (Hey et al. 2018), mstree is faster than 3s and IMa3 (supplementary table S7, Supplementary Material online). Although 3s and IMa3 performed very well on some parameter estimates, the *τ*_1_ estimates of 3s and *τ*_0_ estimates of IMa3 were very poor.

### Robustness of mstree and Analyzing Real Data

Though our method is not affected by gene flow between the sister species, our method assumes that there is no gene flow between the ingroup and the outgroup. Therefore, we examined the robustness of our method in the presence of gene flow between the ingroup and the outgroup. The results are shown in supplementary table S8, Supplementary Material online. Our method is robust to the simulations based on IIM model between the ingroup and the outgroup. For the simulations based on SC and IM model between the ingroup and the outgroup, the accuracy of parameter estimates is on the decline. At last, we apply mstree to the genomic sequences of the human (H), chimpanzee (C), and gorilla (G) (Burgess and Yang 2008). The estimates of parameters are similar to those of Burgess and Yang (2008), but the estimate of *τ*_HC_ is slightly higher (supplementary table S9, Supplementary Material online). In order to quantify uncertainty in the estimates obtained, we resort to bootstrapping with 100 replicates. The averages and the standard errors of estimates are as follows: ${\hat{\theta }}_{\mathrm{HCG}}=0.0032\;\pm \;0.0000$, ${\hat{\theta }}_{\mathrm{HC}}=0.0068\;\pm \;0.0005$, ${\hat{\tau }}_{\mathrm{HCG}}=0.0059\;\pm \;0.0000$, and ${\hat{\tau }}_{\mathrm{HC}}=0.0038\;\pm \;0.0005$.

## Discussion

Supplementary table S8, Supplementary Material online, shows the performance of mstree in the presence of gene flow with species 3. Under the influence of gene flow between the ingroup and the outgroup, *τ*_0_ was underestimated and was closed to the time that gene flow stopped except for IIM model. When *τ*_0_ was underestimated, the estimates of other parameters were far away from the true value. Burgess and Yang (2008) estimated divergence times under the assumption of no gene flow. However, Zhu and Yang (2012) applied the test based on SIM3s model to a human–chimpanzee–gorilla genomic data and the test results suggested gene flow around the time of speciation of human and chimpanzee. Compared with the estimated divergence times from Burgess and Yang (2008), the analysis from mstree suggested migrations between sister species. In addition, there are two significant differences between mstree and COALGF (Tian and Kubatko 2016), which describes the distribution of coalescent histories under the coalescent model with gene flow: 1) mstree uses the coalescent history distribution under coalescent model without gene flow to infer model parameters based on summary statistics; however, COALGF computes probabilities of gene tree histories given species trees under the coalescent process with gene flow and the results obtained from COALGF may be used to infer model parameters based on a maximum likelihood framework. 2) mstree does not make any assumption about the mode of gene flow between sister taxa; however, COALGF assumes that the mode of gene flow between sister taxa is IM.

To summarize, we propose a multispecies coalescent approach, mstree, for estimating the parameters during speciation with gene flow. Theoretically, our method does not rely on any assumption about the relationship between isolation and migration. Furthermore, the simulation results demonstrate that mstree can accurately estimate species divergence time and ancestral population size regardless of IIM model, SC model, or IM model.

## Supplementary Material

evaa087_Supplementary_Data
