# Supplementary material for: mstree: A Multispecies Coalescent Approach for Estimating Ancestral Population Size and Divergence Time during Speciation with Gene Flow
Source: Genome Biol Evol. 2020 May 4;12(5):715–9. doi: 10.1093/gbe/evaa087 (PMC7259675; doi:10.1093/gbe/evaa087)
Supplement: evaa087_Supplementary_Data [file evaa087_supplementary_data.zip › File_S1_Supp_revision_noremark.docx]

Supplementary Material

# mstree: a multispecies coalescent approach for estimating ancestral population size and divergence time during speciation with gene flow

**Simulation Results**

**Table S1.** The estimated species divergence time and population size for Hominoid set when the migration rate is 0.1

|  |  | symmetry | | | | asymmetry | | | |
| --- | --- | --- | --- | --- | --- | --- | --- | --- | --- |
|  | Number of loci | $\theta_{0}$ | $\theta_{1}$ | $\tau_{0}$ | $\tau_{1}$ | $\theta_{0}$ | $\theta_{1}$ | $\tau_{0}$ | $\tau_{1}$ |
| IIM model | 5000 | 0.50$\pm$0.03 | 0.49$\pm$0.04 | 0.60$\pm$0.00 | 0.44$\pm$0.06 | 0.50$\pm$0.05 | 0.51$\pm$0.08 | 0.60$\pm$0.00 | 0.45$\pm$0.08 |
|  | 10,000 | 0.50$\pm$0.02 | 0.49$\pm$0.02 | 0.60$\pm$0.00 | 0.44$\pm$0.06 | 0.50$\pm$0.02 | 0.49$\pm$0.03 | 0.60$\pm$0.00 | 0.43$\pm$0.06 |
|  | 50,000 | 0.50$\pm$0.01 | 0.50$\pm$0.01 | 0.60$\pm$0.00 | 0.41$\pm$0.03 | 0.50$\pm$0.01 | 0.50$\pm$0.01 | 0.60$\pm$0.00 | 0.41$\pm$0.03 |
| SC model | 5000 | 0.50$\pm$0.03 | 0.49$\pm$0.05 | 0.60$\pm$0.00 | 0.45$\pm$0.07 | 0.50$\pm$0.03 | 0.55$\pm$0.12 | 0.60$\pm$0.00 | 0.45$\pm$0.10 |
|  | 10,000 | 0.50$\pm$0.02 | 0.49$\pm$0.03 | 0.60$\pm$0.00 | 0.44$\pm$0.07 | 0.50$\pm$0.02 | 0.49$\pm$0.03 | 0.60$\pm$0.00 | 0.44$\pm$0.07 |
|  | 50,000 | 0.50$\pm$0.01 | 0.50$\pm$0.01 | 0.60$\pm$0.00 | 0.42$\pm$0.04 | 0.50$\pm$0.01 | 0.50$\pm$0.01 | 0.60$\pm$0.00 | 0.42$\pm$0.04 |
| IM model | 5000 | 0.50$\pm$0.03 | 0.48$\pm$0.05 | 0.60$\pm$0.00 | 0.46$\pm$0.07 | 0.50$\pm$0.03 | 0.49$\pm$0.03 | 0.60$\pm$0.00 | 0.45$\pm$0.07 |
|  | 10,000 | 0.50$\pm$0.02 | 0.49$\pm$0.04 | 0.60$\pm$0.00 | 0.44$\pm$0.07 | 0.50$\pm$0.02 | 0.49$\pm$0.03 | 0.60$\pm$0.00 | 0.44$\pm$0.06 |
|  | 50,000 | 0.50$\pm$0.01 | 0.50$\pm$0.01 | 0.60$\pm$0.00 | 0.42$\pm$0.04 | 0.50$\pm$0.01 | 0.50$\pm$0.01 | 0.60$\pm$0.00 | 0.42$\pm$0.04 |

Note. The hominoid set is *θ*_0_=*θ*_1_=0.005, *τ*_0_=0.006 and *τ*_1_=0.004. *θ* and *τ* estimates are scaled by 10^2^. The threshold value in mstree is 0.03. The number of replicates is 1000. IIM, SC, IM models mean isolation-with-initial migration model, secondary contact model and isolation-with-migration model, respectively.

**Table S2.** The estimated species divergence time and population size for Hominoid set when the migration rate is 1

|  |  | symmetry | | | | asymmetry | | | |
| --- | --- | --- | --- | --- | --- | --- | --- | --- | --- |
|  | Number of loci | $\theta_{0}$ | $\theta_{1}$ | $\tau_{0}$ | $\tau_{1}$ | $\theta_{0}$ | $\theta_{1}$ | $\tau_{0}$ | $\tau_{1}$ |
| IIM model | 5000 | 0.50$\pm$0.03 | 0.48$\pm$0.05 | 0.60$\pm$0.00 | 0.44$\pm$0.07 | 0.50$\pm$0.03 | 0.49$\pm$0.04 | 0.60$\pm$0.00 | 0.44$\pm$0.07 |
|  | 10,000 | 0.50$\pm$0.02 | 0.49$\pm$0.03 | 0.60$\pm$0.00 | 0.44$\pm$0.06 | 0.50$\pm$0.02 | 0.49$\pm$0.03 | 0.60$\pm$0.00 | 0.44$\pm$0.07 |
|  | 50,000 | 0.50$\pm$0.01 | 0.50$\pm$0.01 | 0.60$\pm$0.00 | 0.41$\pm$0.04 | 0.50$\pm$0.01 | 0.50$\pm$0.01 | 0.60$\pm$0.00 | 0.41$\pm$0.04 |
| SC model | 5000 | 0.50$\pm$0.02 | 0.48$\pm$0.05 | 0.60$\pm$0.00 | 0.46$\pm$0.07 | 0.50$\pm$0.02 | 0.48$\pm$0.05 | 0.60$\pm$0.01 | 0.46$\pm$0.08 |
|  | 10,000 | 0.50$\pm$0.01 | 0.48$\pm$0.05 | 0.60$\pm$0.00 | 0.45$\pm$0.07 | 0.50$\pm$0.01 | 0.49$\pm$0.04 | 0.60$\pm$0.00 | 0.45$\pm$0.07 |
|  | 50,000 | 0.50$\pm$0.01 | 0.50$\pm$0.01 | 0.60$\pm$0.00 | 0.42$\pm$0.05 | 0.50$\pm$0.01 | 0.50$\pm$0.02 | 0.60$\pm$0.00 | 0.42$\pm$0.06 |
| IM model | 5000 | 0.50$\pm$0.03 | 0.47$\pm$0.07 | 0.60$\pm$0.01 | 0.46$\pm$0.08 | 0.50$\pm$0.04 | 0.48$\pm$0.07 | 0.60$\pm$0.01 | 0.41$\pm$0.11 |
|  | 10,000 | 0.50$\pm$0.01 | 0.49$\pm$0.04 | 0.60$\pm$0.00 | 0.44$\pm$0.07 | 0.50$\pm$0.01 | 0.49$\pm$0.05 | 0.60$\pm$0.00 | 0.41$\pm$0.10 |
|  | 50,000 | 0.50$\pm$0.01 | 0.50$\pm$0.01 | 0.60$\pm$0.00 | 0.42$\pm$0.05 | 0.50$\pm$0.01 | 0.50$\pm$0.02 | 0.60$\pm$0.00 | 0.41$\pm$0.07 |

Note. The hominoid set is *θ*_0_=*θ*_1_=0.005, *τ*_0_=0.006 and *τ*_1_=0.004. *θ* and *τ* estimates are scaled by 10^2^. The threshold value in mstree is 0.03. The number of replicates is 1000. IIM, SC, IM models mean isolation-with-initial migration model, secondary contact model and isolation-with-migration model, respectively.

**Table S3.** The estimated species divergence time and population size for Hominoid set when the migration rate is 10

|  |  | symmetry | | | | asymmetry | | | |
| --- | --- | --- | --- | --- | --- | --- | --- | --- | --- |
|  | Number of loci | $\theta_{0}$ | $\theta_{1}$ | $\tau_{0}$ | $\tau_{1}$ | $\theta_{0}$ | $\theta_{1}$ | $\tau_{0}$ | $\tau_{1}$ |
| IIM model | 5000 | 0.50$\pm$0.02 | 0.49$\pm$0.04 | 0.60$\pm$0.00 | 0.44$\pm$0.07 | 0.50$\pm$0.02 | 0.49$\pm$0.04 | 0.60$\pm$0.00 | 0.37$\pm$0.11 |
|  | 10,000 | 0.50$\pm$0.02 | 0.49$\pm$0.03 | 0.60$\pm$0.00 | 0.44$\pm$0.07 | 0.50$\pm$0.01 | 0.49$\pm$0.03 | 0.60$\pm$0.00 | 0.35$\pm$0.09 |
|  | 50,000 | 0.50$\pm$0.01 | 0.50$\pm$0.01 | 0.60$\pm$0.00 | 0.41$\pm$0.04 | 0.50$\pm$0.01 | 0.50$\pm$0.01 | 0.60$\pm$0.00 | 0.31$\pm$0.06 |
| SC model | 5000 | 0.50$\pm$0.04 | 0.48$\pm$0.06 | 0.60$\pm$0.01 | 0.46$\pm$0.07 | 0.50$\pm$0.02 | 0.47$\pm$0.07 | 0.60$\pm$0.01 | 0.36$\pm$0.18 |
|  | 10,000 | 0.50$\pm$0.01 | 0.49$\pm$0.04 | 0.60$\pm$0.00 | 0.45$\pm$0.07 | 0.50$\pm$0.01 | 0.48$\pm$0.05 | 0.60$\pm$0.01 | 0.30$\pm$0.19 |
|  | 50,000 | 0.50$\pm$0.01 | 0.50$\pm$0.01 | 0.60$\pm$0.00 | 0.42$\pm$0.05 | 0.50$\pm$0.01 | 0.50$\pm$0.01 | 0.60$\pm$0.00 | 0.19$\pm$0.18 |
| IM model | 5000 | 0.50$\pm$0.02 | 0.47$\pm$0.07 | 0.60$\pm$0.01 | 0.46$\pm$0.08 | 0.50$\pm$0.02 | 0.47$\pm$0.07 | 0.60$\pm$0.01 | 0.34$\pm$0.18 |
|  | 10,000 | 0.50$\pm$0.02 | 0.49$\pm$0.04 | 0.60$\pm$0.00 | 0.45$\pm$0.07 | 0.50$\pm$0.01 | 0.48$\pm$0.05 | 0.60$\pm$0.01 | 0.30$\pm$0.19 |
|  | 50,000 | 0.50$\pm$0.01 | 0.50$\pm$0.02 | 0.60$\pm$0.00 | 0.42$\pm$0.05 | 0.50$\pm$0.01 | 0.50$\pm$0.01 | 0.60$\pm$0.00 | 0.20$\pm$0.19 |

Note. The hominoid set is *θ*_0_=*θ*_1_=0.005, *τ*_0_=0.006 and *τ*_1_=0.004. *θ* and *τ* estimates are scaled by 10^2^. The threshold value in mstree is 0.03. The number of replicates is 1000. IIM, SC, IM models mean isolation-with-initial migration model, secondary contact model and isolation-with-migration model, respectively.

**Table S4.** The estimated species divergence time and population size for Mangrove set when the migration rate is 0.1

|  |  | symmetry | | | | asymmetry | | | |
| --- | --- | --- | --- | --- | --- | --- | --- | --- | --- |
|  | Number of loci | $\theta_{0}$ | $\theta_{1}$ | $\tau_{0}$ | $\tau_{1}$ | $\theta_{0}$ | $\theta_{1}$ | $\tau_{0}$ | $\tau_{1}$ |
| IIM model | 5000 | 1.00$\pm$0.03 | 0.99$\pm$0.03 | 2.00$\pm$0.01 | 1.09$\pm$0.22 | 1.00$\pm$0.03 | 0.99$\pm$0.03 | 2.00$\pm$0.01 | 1.07$\pm$0.20 |
|  | 10,000 | 1.00$\pm$0.02 | 0.99$\pm$0.02 | 2.00$\pm$0.01 | 1.03$\pm$0.14 | 1.00$\pm$0.02 | 1.00$\pm$0.02 | 2.00$\pm$0.01 | 1.03$\pm$0.14 |
|  | 50,000 | 1.00$\pm$0.01 | 1.00$\pm$0.01 | 2.00$\pm$0.00 | 1.00$\pm$0.04 | 1.00$\pm$0.01 | 1.00$\pm$0.01 | 2.00$\pm$0.00 | 1.00$\pm$0.03 |
| SC model | 5000 | 1.00$\pm$0.03 | 0.99$\pm$0.04 | 2.00$\pm$0.02 | 1.09$\pm$0.22 | 1.00$\pm$0.03 | 0.99$\pm$0.04 | 2.00$\pm$0.01 | 1.07$\pm$0.20 |
|  | 10,000 | 1.00$\pm$0.02 | 0.99$\pm$0.02 | 2.00$\pm$0.01 | 1.05$\pm$0.16 | 1.00$\pm$0.02 | 0.99$\pm$0.02 | 2.00$\pm$0.01 | 1.04$\pm$0.13 |
|  | 50,000 | 1.00$\pm$0.01 | 1.00$\pm$0.01 | 2.00$\pm$0.00 | 1.00$\pm$0.05 | 1.00$\pm$0.01 | 1.00$\pm$0.01 | 2.00$\pm$0.00 | 1.00$\pm$0.04 |
| IM model | 5000 | 1.00$\pm$0.03 | 0.99$\pm$0.04 | 2.00$\pm$0.02 | 1.10$\pm$0.24 | 1.00$\pm$0.03 | 0.99$\pm$0.03 | 2.00$\pm$0.02 | 1.08$\pm$0.22 |
|  | 10,000 | 1.00$\pm$0.02 | 0.99$\pm$0.02 | 2.00$\pm$0.01 | 1.06$\pm$0.18 | 1.00$\pm$0.02 | 0.99$\pm$0.02 | 2.00$\pm$0.01 | 1.05$\pm$0.17 |
|  | 50,000 | 1.00$\pm$0.01 | 1.00$\pm$0.01 | 2.00$\pm$0.01 | 1.00$\pm$0.05 | 1.00$\pm$0.01 | 1.00$\pm$0.01 | 2.00$\pm$0.01 | 1.00$\pm$0.06 |

Note. The mangrove set is *θ*_0_=*θ*_1_=0.01, *τ*_0_=0.02 and *τ*_1_=0.01. *θ* and *τ* estimates are scaled by 10^2^. The threshold value in mstree is 0.03. The number of replicates is 1000. IIM, SC, IM models mean isolation-with-initial migration model, secondary contact model and isolation-with-migration model, respectively.

**Table S5.** The estimated species divergence time and population size for Mangrove set when the migration rate is 1

|  |  | symmetry | | | | asymmetry | | | |
| --- | --- | --- | --- | --- | --- | --- | --- | --- | --- |
|  | Number of loci | $\theta_{0}$ | $\theta_{1}$ | $\tau_{0}$ | $\tau_{1}$ | $\theta_{0}$ | $\theta_{1}$ | $\tau_{0}$ | $\tau_{1}$ |
| IIM model | 5000 | 1.00$\pm$0.03 | 0.99$\pm$0.03 | 2.00$\pm$0.02 | 1.08$\pm$0.22 | 1.00$\pm$0.03 | 0.99$\pm$0.05 | 2.00$\pm$0.02 | 1.07$\pm$0.23 |
|  | 10,000 | 1.00$\pm$0.02 | 0.99$\pm$0.03 | 2.00$\pm$0.01 | 1.04$\pm$0.16 | 1.00$\pm$0.02 | 0.99$\pm$0.02 | 2.00$\pm$0.01 | 1.02$\pm$0.17 |
|  | 50,000 | 1.00$\pm$0.01 | 1.00$\pm$0.01 | 2.00$\pm$0.01 | 0.99$\pm$0.05 | 1.00$\pm$0.01 | 1.00$\pm$0.01 | 2.00$\pm$0.01 | 0.98$\pm$0.06 |
| SC model | 5000 | 1.00$\pm$0.03 | 0.98$\pm$0.05 | 2.00$\pm$0.02 | 1.11$\pm$0.24 | 1.00$\pm$0.03 | 0.98$\pm$0.07 | 2.00$\pm$0.02 | 1.12$\pm$0.27 |
|  | 10,000 | 1.00$\pm$0.02 | 0.99$\pm$0.03 | 2.00$\pm$0.01 | 1.06$\pm$0.19 | 1.00$\pm$0.02 | 0.99$\pm$0.03 | 2.00$\pm$0.02 | 1.09$\pm$0.23 |
|  | 50,000 | 1.00$\pm$0.01 | 1.00$\pm$0.01 | 2.00$\pm$0.01 | 1.00$\pm$0.07 | 1.00$\pm$0.01 | 1.00$\pm$0.01 | 2.00$\pm$0.01 | 1.01$\pm$0.10 |
| IM model | 5000 | 1.00$\pm$0.04 | 0.98$\pm$0.05 | 2.00$\pm$0.02 | 1.13$\pm$0.27 | 1.00$\pm$0.03 | 0.97$\pm$0.08 | 2.00$\pm$0.03 | 1.04$\pm$0.39 |
|  | 10,000 | 1.00$\pm$0.02 | 0.99$\pm$0.03 | 2.00$\pm$0.02 | 1.08$\pm$0.22 | 1.00$\pm$0.02 | 0.98$\pm$0.06 | 2.00$\pm$0.02 | 0.98$\pm$0.31 |
|  | 50,000 | 1.00$\pm$0.01 | 1.00$\pm$0.01 | 2.00$\pm$0.01 | 1.00$\pm$0.08 | 1.00$\pm$0.01 | 1.00$\pm$0.02 | 2.00$\pm$0.01 | 0.89$\pm$0.17 |

Note. The mangrove set is *θ*_0_=*θ*_1_=0.01, *τ*_0_=0.02 and *τ*_1_=0.01. *θ* and *τ* estimates are scaled by 10^2^. The threshold value in mstree is 0.03. The number of replicates is 1000. IIM, SC, IM models mean isolation-with-initial migration model, secondary contact model and isolation-with-migration model, respectively.

**Table S6.** The estimated species divergence time and population size for Mangrove set when the migration rate is 10

|  |  | symmetry | | | | asymmetry | | | |
| --- | --- | --- | --- | --- | --- | --- | --- | --- | --- |
|  | Number of loci | $\theta_{0}$ | $\theta_{1}$ | $\tau_{0}$ | $\tau_{1}$ | $\theta_{0}$ | $\theta_{1}$ | $\tau_{0}$ | $\tau_{1}$ |
| IIM model | 5000 | 1.00$\pm$0.04 | 0.99$\pm$0.03 | 2.00$\pm$0.02 | 1.08$\pm$0.22 | 1.00$\pm$0.03 | 0.98$\pm$0.04 | 2.00$\pm$0.02 | 0.91$\pm$0.32 |
|  | 10,000 | 1.00$\pm$0.02 | 0.99$\pm$0.02 | 2.00$\pm$0.01 | 1.02$\pm$0.13 | 1.00$\pm$0.02 | 0.99$\pm$0.03 | 2.00$\pm$0.01 | 0.83$\pm$0.27 |
|  | 50,000 | 1.00$\pm$0.01 | 1.00$\pm$0.01 | 2.00$\pm$0.01 | 0.99$\pm$0.04 | 1.00$\pm$0.01 | 1.00$\pm$0.01 | 2.00$\pm$0.01 | 0.73$\pm$0.12 |
| SC model | 5000 | 1.00$\pm$0.03 | 0.98$\pm$0.05 | 2.00$\pm$0.02 | 1.11$\pm$0.24 | 1.00$\pm$0.03 | 0.95$\pm$0.12 | 1.99$\pm$0.04 | 0.98$\pm$0.56 |
|  | 10,000 | 1.00$\pm$0.02 | 0.99$\pm$0.04 | 2.00$\pm$0.01 | 1.08$\pm$0.21 | 1.00$\pm$0.02 | 0.98$\pm$0.07 | 2.00$\pm$0.03 | 0.84$\pm$0.56 |
|  | 50,000 | 1.00$\pm$0.01 | 1.00$\pm$0.01 | 2.00$\pm$0.01 | 1.01$\pm$0.08 | 1.00$\pm$0.01 | 0.99$\pm$0.02 | 2.00$\pm$0.01 | 0.53$\pm$0.51 |
| IM model | 5000 | 1.00$\pm$0.04 | 0.98$\pm$0.07 | 2.00$\pm$0.02 | 1.13$\pm$0.27 | 1.00$\pm$0.03 | 0.95$\pm$0.13 | 2.00$\pm$0.04 | 1.01$\pm$0.57 |
|  | 10,000 | 1.00$\pm$0.02 | 0.99$\pm$0.03 | 2.00$\pm$0.02 | 1.08$\pm$0.23 | 1.00$\pm$0.02 | 0.97$\pm$0.08 | 2.00$\pm$0.03 | 0.86$\pm$0.57 |
|  | 50,000 | 1.00$\pm$0.01 | 1.00$\pm$0.01 | 2.00$\pm$0.01 | 1.01$\pm$0.11 | 1.00$\pm$0.01 | 0.99$\pm$0.03 | 2.00$\pm$0.01 | 0.59$\pm$0.53 |

Note. The mangrove set is *θ*_0_=*θ*_1_=0.01, *τ*_0_=0.02 and *τ*_1_=0.01. *θ* and *τ* estimates are scaled by 10^2^. The threshold value in mstree is 0.03. The number of replicates is 1000. IIM, SC, IM models mean isolation-with-initial migration model, secondary contact model and isolation-with-migration model, respectively.

**Table S7.** The estimated species divergence time and population size for Mangrove set with the effect of the gene tree uncertainty and compared mstree with 3s and IMa3

|  | Program | $\theta_{0}$ | $\theta_{1}$ | $\tau_{0}$ | $\tau_{1}$ | Time used |
| --- | --- | --- | --- | --- | --- | --- |
| IIM model | mstree | 1.28$\pm$0.23 | 1.04$\pm$0.02 | 1.97$\pm$0.02 | 1.06$\pm$0.21 | about 1min |
|  | 3s | 0.98$\pm$0.02 | 1.07$\pm$0.02 | 1.99$\pm$0.00 | 0.82$\pm$0.00 | about 39min |
|  | IMa3 | 1.00$\pm$0.05 | 1.00$\pm$0.05 | 2.67$\pm$0.04 | 1.33$\pm$0.05 | about 6min |
| SC model | mstree | 1.21$\pm$0.20 | 1.06$\pm$0.03 | 1.97$\pm$0.02 | 1.06$\pm$0.21 | about 1min |
|  | 3s | 0.96$\pm$0.02 | 1.86$\pm$0.19 | 2.00$\pm$0.00 | 0.11$\pm$0.21 | about 39min |
|  | IMa3 | 1.00$\pm$0.04 | 1.00$\pm$0.03 | 2.67$\pm$0.03 | 1.33$\pm$0.05 | about 6min |
| IM model | mstree | 1.15$\pm$0.17 | 1.03$\pm$0.03 | 1.97$\pm$0.03 | 1.02$\pm$0.14 | about 1min |
|  | 3s | 0.96$\pm$0.02 | 1.65$\pm$0.13 | 2.00$\pm$0.00 | 0.12$\pm$0.17 | about 39min |
|  | IMa3 | 1.00$\pm$0.05 | 1.01$\pm$0.03 | 2.66$\pm$0.04 | 1.33$\pm$0.04 | about 6min |

Note. The mangrove set is *θ*_0_=*θ*_1_=0.01, *τ*_0_=0.02 and *τ*_1_=0.01. *θ* and *τ* estimates are scaled by 10^2^. Gene flow is symmetrical and the migration rate is 1. The threshold value in mstree is 0.03. The length of sequence at each locus is 500bp. For mstree and 3s, the number of loci is 10 000. For IMa3, the number of loci is 100. The number of replicates is 100. IIM, SC, IM models mean isolation-with-initial migration model, secondary contact model and isolation-with-migration model, respectively.

**Table S8.** The estimated species divergence time and population size in presence of gene flow between the ingroup and the outgroup

|  |  | Hominoid | | | | Mangrove | | | |
| --- | --- | --- | --- | --- | --- | --- | --- | --- | --- |
| ModeA | ModeB | $\theta_{0}$ | $\theta_{1}$ | $\tau_{0}$ | $\tau_{1}$ | $\theta_{0}$ | $\theta_{1}$ | $\tau_{0}$ | $\tau_{1}$ |
| IIM model | IIM | **0.54**$\boldsymbol{\pm}$**0.04** | **0.49**$\boldsymbol{\pm}$**0.02** | **0.56**$\boldsymbol{\pm}$**0.00** | **0.39**$\boldsymbol{\pm}$**0.01** | **1.00**$\boldsymbol{\pm}$**0.02** | **0.98**$\boldsymbol{\pm}$**0.02** | **1.74**$\boldsymbol{\pm}$**0.01** | **0.99**$\boldsymbol{\pm}$**0.04** |
|  | SC | 0.50$\pm$0.03 | 0.43$\pm$0.03 | 0.45$\pm$0.00 | 0.39$\pm$0.01 | 1.28$\pm$0.50 | 0.92$\pm$0.10 | 1.07$\pm$0.00 | 0.97$\pm$0.03 |
|  | IM | 0.50$\pm$0.03 | 0.46$\pm$0.05 | 0.43$\pm$0.00 | 0.38$\pm$0.01 | 1.99$\pm$0.36 | 0.93$\pm$0.13 | 1.07$\pm$0.01 | 0.96$\pm$0.04 |
| SC model | IIM | **0.53**$\boldsymbol{\pm}$**0.04** | **0.48**$\boldsymbol{\pm}$**0.02** | **0.56**$\boldsymbol{\pm}$**0.00** | **0.39**$\boldsymbol{\pm}$**0.01** | **1.00**$\boldsymbol{\pm}$**0.02** | **0.98**$\boldsymbol{\pm}$**0.02** | **1.74**$\boldsymbol{\pm}$**0.02** | **1.00**$\boldsymbol{\pm}$**0.05** |
|  | SC | 0.50$\pm$0.02 | 0.56$\pm$0.26 | 0.45$\pm$0.00 | 0.32$\pm$0.14 | 1.22$\pm$0.46 | 2.45$\pm$0.43 | 1.07$\pm$0.01 | 0.23$\pm$0.27 |
|  | IM | 0.50$\pm$0.02 | 1.04$\pm$0.26 | 0.43$\pm$0.00 | 0.10$\pm$0.14 | 2.04$\pm$0.33 | 2.49$\pm$0.41 | 1.07$\pm$0.01 | 0.23$\pm$0.26 |
| IM model | IIM | **0.54**$\boldsymbol{\pm}$**0.04** | **0.48**$\boldsymbol{\pm}$**0.02** | **0.56**$\boldsymbol{\pm}$**0.01** | **0.39**$\boldsymbol{\pm}$**0.01** | **1.00**$\boldsymbol{\pm}$**0.02** | **0.98**$\boldsymbol{\pm}$**0.03** | **1.74**$\boldsymbol{\pm}$**0.02** | **0.99**$\boldsymbol{\pm}$**0.05** |
|  | SC | 0.50$\pm$0.02 | 0.50$\pm$0.16 | 0.45$\pm$0.01 | 0.35$\pm$0.09 | 1.18$\pm$0.43 | 1.60$\pm$0.34 | 1.07$\pm$0.01 | 0.51$\pm$0.25 |
|  | IM | 0.50$\pm$0.02 | 0.76$\pm$0.18 | 0.43$\pm$0.00 | 0.21$\pm$0.11 | 2.06$\pm$0.31 | 1.61$\pm$0.35 | 1.07$\pm$0.01 | 0.49$\pm$0.26 |

Note. The hominoid set is *θ*_0_=*θ*_1_=0.005, *τ*_0_=0.006 and *τ*_1_=0.004. The mangrove set is *θ*_0_=*θ*_1_=0.01, *τ*_0_=0.02 and *τ*_1_=0.01. *θ* and *τ* estimates are scaled by 10^2^. Gene flow is symmetrical and the migration rate is 1. The threshold value in mstree is 0.03. The number of loci is 10 000. The number of replicates is 1000. ModeA denotes the mode of gene flow between sister species; ModeB denotes the mode of gene flow between the ingroup and the outgroup. IIM, SC, IM models mean isolation-with-initial migration model, secondary contact model and isolation-with-migration model, respectively. The best estimates are marked in bold.

**Table S9.** The estimated species divergence time and population size for Hominoid Genomic Loci

|  | $\theta_{\mathrm{HCG}}$ | $\theta_{\mathrm{HC}}$ | $\tau_{\mathrm{HCG}}$ | $\tau_{\mathrm{HC}}$ |
| --- | --- | --- | --- | --- |
| mstree | 0.32 | 0.66 | 0.59 | 0.40 |
| BY08^a^ | 0.35 | 0.63 | 0.59 | 0.37 |
| mstree^b^ | 0.32$\pm$0.00 | 0.68$\pm$0.05 | 0.59$\pm$0.00 | 0.38$\pm$0.05 |

Note. *θ* and *τ* estimates are scaled by 10^2^. The data set comprises 14,663 autosomal loci and the mean locus length is 508bp. The program dnamlk in PHYLIP package was used to infer gene trees.

^a^ The posterior means from Burgess and Yang (2008: table 2).

^b^ The bootstrapping estimates with 100 replicates.


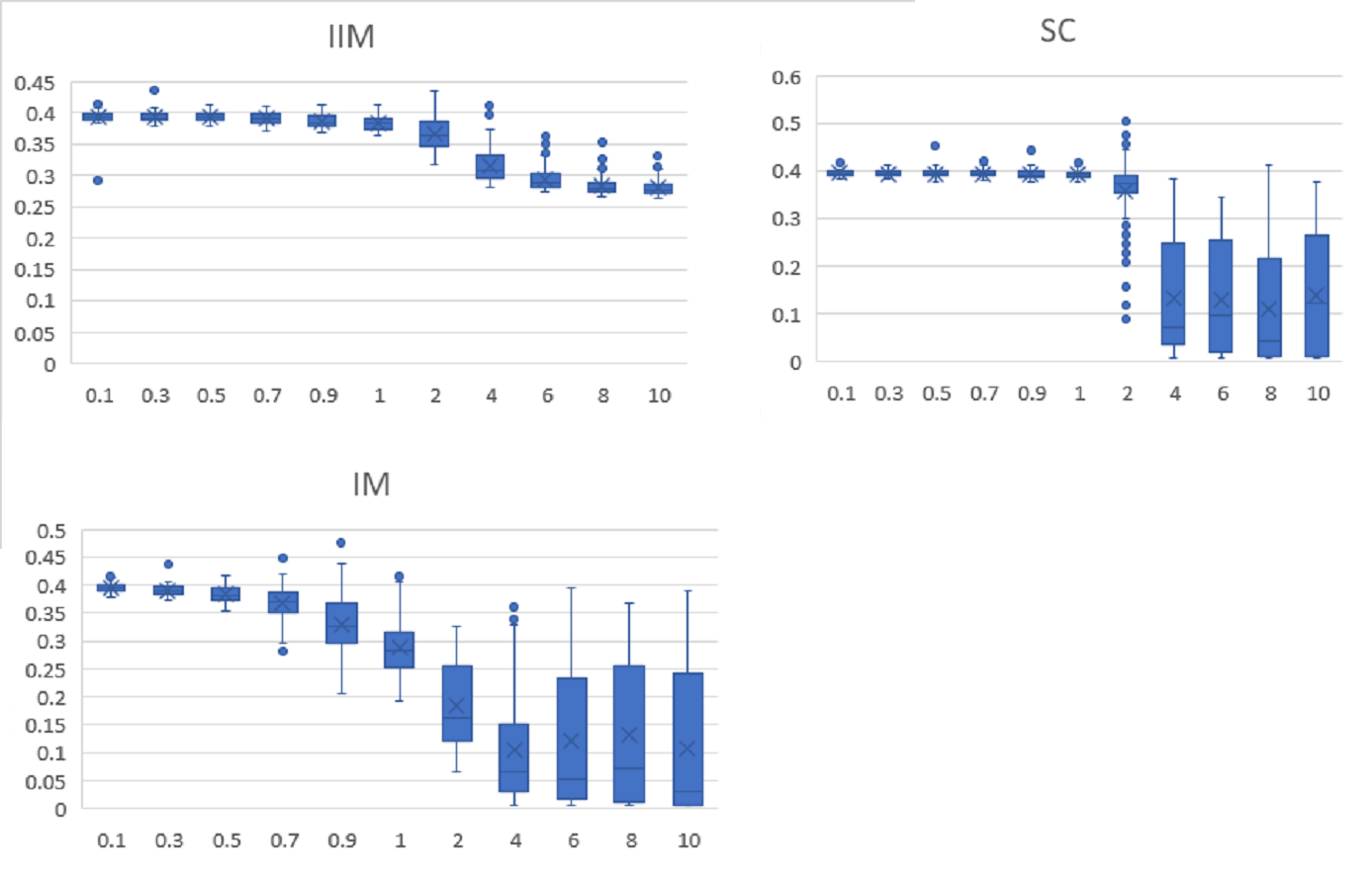


Fig. S1.­—The trend of the estimated τ_1_ with increasing asymmetry migration rate. The true value is 0.004 and the estimates are scaled by 10^2^. Gene flow is symmetrical and the migration rate varies from 0.1 to 10. The threshold value in mstree is 0.03. The number of loci is 10 000. The number of replicates is 100. IIM, SC, IM models mean isolation-with-initial migration model, secondary contact model and isolation-with-migration model, respectively.
