# Supplementary material for: mstree: A Multispecies Coalescent Approach for Estimating Ancestral Population Size and Divergence Time during Speciation with Gene Flow
Source: Genome Biol Evol. 2020 May 4;12(5):715–9. doi: 10.1093/gbe/evaa087 (PMC7259675; doi:10.1093/gbe/evaa087)
Supplement: evaa087_Supplementary_Data [file evaa087_supplementary_data.zip › File_S2_Supp_revision_noremark.docx]

Supplementary Material

# mstree: a multispecies coalescent approach for estimating ancestral population size and divergence time during speciation with gene flow

**Simulation Commands**

--IIM model (hominoids)

./ms 3 5000 -t 0.005 -T -I 3 1 1 1 -m 1 2 0 -m 2 1 0 -em 0.533 1 2 0.4 -em 0.533 2 1 0.4 -em 0.8 1 2 0 -em 0.8 2 1 0 -ej 0.8 2 1 -ej 1.2 3 1 | tail -n +4 | grep -v // > tree

Note. The number of loci is 5000 and migration rate is 0.1 with symmetrical gene flow.

./ms 3 5000 -t 0.005 -T -I 3 1 1 1 -m 1 2 0 -m 2 1 0 -em 0.533 1 2 0.4 -em 0.533 2 1 0 -em 0.8 1 2 0 -em 0.8 2 1 0 -ej 0.8 2 1 -ej 1.2 3 1 | tail -n +4 | grep -v // > tree

Note. The number of loci is 5000 and migration rate is 0.1 with asymmetrical gene flow.

--SC model (hominoids)

./ms 3 5000 -t 0.005 -T -I 3 1 1 1 -m 1 2 0.4 -m 2 1 0.4 -em 0.267 1 2 0 -em 0.267 2 1 0 -ej 0.8 2 1 -ej 1.2 3 1 | tail -n +4 | grep -v // > tree

Note. The number of loci is 5000 and migration rate is 0.1 with symmetrical gene flow.

./ms 3 5000 -t 0.005 -T -I 3 1 1 1 -m 1 2 0.4 -m 2 1 0 -em 0.267 1 2 0 -em 0.267 2 1 0 -ej 0.8 2 1 -ej 1.2 3 1 | tail -n +4 | grep -v // > tree

Note. The number of loci is 5000 and migration rate is 0.1 with asymmetrical gene flow.

--IM model (hominoids)

./ms 3 5000 -t 0.005 -T -I 3 1 1 1 -m 1 2 0.4 -m 2 1 0.4 -em 0.8 1 2 0 -em 0.8 2 1 0 -ej 0.8 2 1 -ej 1.2 3 1 | tail -n +4 | grep -v // > tree

Note. The number of loci is 5000 and migration rate is 0.1 with symmetrical gene flow.

./ms 3 5000 -t 0.005 -T -I 3 1 1 1 -m 1 2 0.4 -m 2 1 0 -em 0.8 1 2 0 -em 0.8 2 1 0 -ej 0.8 2 1 -ej 1.2 3 1 | tail -n +4 | grep -v // > tree

Note. The number of loci is 5000 and migration rate is 0.1 with asymmetrical gene flow.

--IIM model (mangroves)

./ms 3 10000 -t 0.01 -T -I 3 1 1 1 -m 1 2 0 -m 2 1 0 -em 0.667 1 2 4 -em 0.667 2 1 4 -em 1 1 2 0 -em 1 2 1 0 -ej 1 2 1 -ej 2 3 1 | tail -n +4 | grep -v // > tree

Note. The number of loci is 10 000 and migration rate is 1 with symmetrical gene flow.

./ms 3 10000 -t 0.01 -T -I 3 1 1 1 -m 1 2 0 -m 2 1 0 -em 0.667 1 2 4 -em 0.667 2 1 0 -em 1 1 2 0 -em 1 2 1 0 -ej 1 2 1 -ej 2 3 1 | tail -n +4 | grep -v // > tree

Note. The number of loci is 10 000 and migration rate is 1 with asymmetrical gene flow.

--SC model (mangroves)

./ms 3 10000 -t 0.01 -T -I 3 1 1 1 -m 1 2 4 -m 2 1 4 -em 0.333 1 2 0 -em 0.333 2 1 0 -ej 1 2 1 -ej 2 3 1 | tail -n +4 | grep -v // > tree

Note. The number of loci is 10 000 and migration rate is 1 with symmetrical gene flow.

./ms 3 10000 -t 0.01 -T -I 3 1 1 1 -m 1 2 4 -m 2 1 0 -em 0.333 1 2 0 -em 0.333 2 1 0 -ej 1 2 1 -ej 2 3 1 | tail -n +4 | grep -v // > tree

Note. The number of loci is 10 000 and migration rate is 1 with asymmetrical gene flow.

--IM model (mangroves)

./ms 3 10000 -t 0.01 -T -I 3 1 1 1 -m 1 2 4 -m 2 1 4 -em 1 1 2 0 -em 1 2 1 0 -ej 1 2 1 -ej 2 3 1 | tail -n +4 | grep -v // > tree

Note. The number of loci is 10 000 and migration rate is 1 with symmetrical gene flow.

./ms 3 10000 -t 0.01 -T -I 3 1 1 1 -m 1 2 4 -m 2 1 0 -em 1 1 2 0 -em 1 2 1 0 -ej 1 2 1 -ej 2 3 1 | tail -n +4 | grep -v // > tree

Note. The number of loci is 10 000 and migration rate is 1 with asymmetrical gene flow.

--inferring gene tree by using dnamlk under IIM model (mangroves)

./ms 3 50000 -T -I 3 1 1 1 -m 1 2 0 -m 2 1 0 -em 0.667 1 2 4 -em 0.667 2 1 4 -em 1 1 2 0 -em 1 2 1 0 -ej 1 2 1 -ej 2 3 1 | tail -n +4 | grep -v // > tree

./seq-gen -m HKY -l 500 -s 0.01 -t 2.0 < tree > infile

echo -e "M\nD\n50000\n123\n3\nY" | ./dnamlk

Note. The length of sequence at each locus is 500bp. The number of loci is 50 000 and migration rate is 1 with symmetrical gene flow.

--IIM_IIM model (hominoids)

./ms 3 10000 -t 0.005 -T -I 3 1 1 1 -m 1 2 0 -m 2 1 0 -em 0.533 1 2 4 -em 0.533 2 1 4 -em 0.8 1 2 0 -em 0.8 2 1 0 -ej 0.8 2 1 -em 1.067 1 3 4 -em 1.067 3 1 4 -em 1.2 1 3 0 -em 1.2 3 1 0 -ej 1.2 3 1 | tail -n +4 | grep -v // > tree

Note. There is gene flow between ingroup and outgroup. The number of loci is 10 000 and migration rate is 1 with symmetrical gene flow.

--IIM_SC model (mangroves)

./ms 3 10000 -t 0.01 -T -I 3 1 1 1 -m 1 2 0 -m 2 1 0 -em 0.667 1 2 4 -em 0.667 2 1 4 -em 1 1 2 0 -em 1 2 1 0 -ej 1 2 1 -em 1 1 3 4 -em 1 3 1 4 -em 1.333 1 3 0 -em 1.333 3 1 0 -ej 2 3 1 | tail -n +4 | grep -v // > tree

Note. There is gene flow between ingroup and outgroup. The number of loci is 10 000 and migration rate is 1 with symmetrical gene flow.
